# Editorial: Impact of new therapeutic approaches for the treatment of post-transplant tumors on the outcome of the transplanted organ

**DOI:** 10.3389/fimmu.2026.1978971

**Published:** 2026-10-22

**Authors:** Piergiorgio Messa, Carlo Maria Alfieri, Josep M. Grinyó

**Affiliations:** 1 Fondazione Ca’ Granda-IRCCS Policlinico, Milano, Italy; 2 Università degli Studi di Milano, Fondazione Ca’ Granda-IRCCS Policlinico, Milano, Italy; 3 Emeritus Professor of Medicine-University of Barcelona, Barcelona, Spain

**Keywords:** immunosuppressive therapy, new therapeutic approaches, post-transplant tumors, solid organ transplant (SOT), transplanted organ

Despite unprecedented improvements in the clinical outcomes of solid organ transplant (SOT) patients in recent decades, these patients continue to have a higher incidence of post-transplant malignancies (PTMs) compared to the general population.

Recent advances in oncology therapies have significantly improved the prognosis of numerous malignancies in the general population. This has sparked considerable interest in the potential application of these new drugs in the clinical setting of solid organ transplantation (SOT). However, many of these new drugs have proven problematic for patients undergoing SOT, due to both organ-specific toxicities and potential interference with immunosuppressive therapy (IST), with consequent repercussions on both the graft and patient outcomes.

Given the gaps in knowledge regarding the management of PTM in solid organ transplant patients, we deemed it useful to launch an editorial initiative to gather contributions aimed at exploring the critical aspects of integrating new therapeutic approaches for PTM.

We have collected a series of very interesting articles, briefly summarized below.

Hiramitsu et al. address the issue of PTM in kidney transplant (KT) patients. Cancer incidence has been reported to be approximately 3-fold higher in solid organ transplant (SOT) patients compared to the general population and is responsible for 20% of post-KT deaths. Induction with ATG and alemtuzumab and higher doses of calcineurin inhibitors (CNIs) have been associated with an increased risk of PTM, while mTOR inhibitors may protect against non-melanoma skin cancers (NMSCs). Other pro-oncogenic factors have been associated with recipient and donor characteristics and environmental factors. Finally, the authors highlight the still limited evidence regarding the use of novel therapeutic approaches for PTM in SOT patients.

Alfieri et al. highlight the lack of defined selection criteria for renal transplant candidates to assess their risk of PTM, highlighting the need for close collaboration between the transplant team and oncologists. The authors confirm that heavier immunosuppressive therapy is associated with an increased risk of PTM, while there is still no definitive evidence of an anti-oncogenic role of mTOR inhibitors. The authors highlight the risk in using belatacept in EBV-seronegative patients, due to an increased risk of post-transplant lymphoproliferative disease (PTLD). After the onset of PTM, the authors recommend reducing the overall burden of immunosuppressive therapy by switching to mTOR inhibitors in patients who develop NMSC. The authors, addressing the issues related to the use of new oncological therapies, summarize their suggestions in a table.

Hsieh et al. reported an incidence of PTM of 11.7% in a cohort of 1,808 renal transplant recipients, with urothelial and liver cancers being the most prevalent tumors. The median latency from KT to PTM diagnosis was 8.2 years (IQR 3.8–12.3)KT, and the median survival was 16.5 months, after cancer diagnosis. Advanced age was the main predictor of PTM onset and mortality. In this cohort, the authors also reported that higher long-exposure to tacrolimus levels were associated with increased mortality. This study highlights that tumor type, timing of onset, IST dependence, and related outcome may vary across renal transplant populations, emphasizing the need to also take geographic and ethnic characteristics into account.

Hiramitsu et al. derive information on the cancer risk in 1274 renal transplants from living donors, of whom 141 developed cancer. The recipient age was the main risk factor, with no significant differences for the type and dose of IST. The onset of cancer was associated with a shorter patient survival, deaths occurring mainly with a functioning transplant. Importantly, the survival of patients with cancer discovered through screening was significantly better than that of patients with cancer diagnosed after the onset of symptoms, highlighting the need for close surveillance.

Dello Strologo et al. deal with PTLD, one of the most frequent PTMs, emphasizing the fundamental role of EBV infection in the onset of PTLD. The authors describe the mechanisms through which EBV-infected B cells shape an immunotolerant tumor microenvironment (TME) that favours the onset and progression of PTLD. The authors also describe the pathogenetic mechanisms of non-EBV-related PTLD, where STI load plays a crucial role in determining the genomic instability that triggers PTLD. The therapeutic options are also discussed, including: STI load reduction, anti-CD20 antibodies and cellular therapies (infusion of EBV-specific cytotoxic T lymphocytes; CAR-T cells). The authors also give the latest information on the use of immune checkpoint inhibitors (ICIs), epigenetic drugs, and pro-apoptotic agents in PTLD.

Gerard et al. addressed the question of whether all classes of ICIs have comparable effects in promoting acute rejection (AR) using data from VigiBase (the WHO safety database containing post-marketing pharmacovigilance data from 172 countries). The authors compared the number of AR cases in patients undergoing SOT treated with ICIs, considered as a whole or as a single class (CTLA-4, PD1 and PDL1 inhibitors), with the number of AR cases associated with the use of other drugs. ICIs have been associated with an increased risk of AR, which occurs within a few weeks of starting ICI therapy. Furthermore, PD-L1 inhibitors appear to be less prone to inducing AR than other classes of ICIs, calling for future studies to confirm these interesting findings.

Mularoni et al. provide a comprehensive review of Kaposi’s sarcoma (KS), which is 400 times more frequent in SOT recipients than in the general population, burdened by a high mortality (35-40%). The prevalence of KS follows the prevalence of HHV-8 seropositivity and is twice as high in HIV seronegative recipients receiving SOT from a HIV seropositive donor compared to seropositive recipients, regardless of the donor’s serologic status. Only 8% of transplant centers perform serologic screening for HHV-8 in both donors and recipients. Despite limited evidence, the authors suggest as a therapeutic approach an early reduction of IST doses, possibly switching from calcineurin inhibitors to mTOR inhibitors, surgical or other local therapies for manifestations limited to the skin, and chemotherapy for the most widespread and aggressive forms. There are no consistent data on the use of ICIs or other potentially useful strategies in the setting of KS in SOT.

We invite all readers of this short editorial to delve deeper into the topics covered in the articles contained in this special issue of Frontiers in Immunology, in the hope that reading these manuscripts will provide useful and stimulating information for future research in a field that still awaits solid scientific evidence.

